# Supplementary material for: Chromothripsis during telomere crisis is independent of NHEJ, and consistent with a replicative origin
Source: Genome Res. 2019 May;29(5):737–49. doi: 10.1101/gr.240705.118 (PMC6499312; doi:10.1101/gr.240705.118)
Supplement: Supplemental Material [file supp_gr.240705.118_Supplemental_file_1.zip › contigs/annotated_contigs/DB73/contig.2.DB73_length_283_mean_cov_3.09187279152.docx]

**DB73_length_283_mean_cov_3.09187279152**

```
GGAAGTAACTTAACCATGGGGAAATAGGCACGGGTACCCCGATGCCTGGCGGGGTTGTGGTTCTTTCTTTCTTTTCTTTTCTCCTTCTT
 >chr16:27899638-27899817 + E=2e-72 p=0e+00
TCTTTCTTTCTTTCTTTCTTTCTTTCTTTCTTTCTTTCTCTCTCTCTTTCTCTCTCTCTCTCTCTTTCTTTCTTTCTTTCT|CCG|TCT
                                                                                     >c
CTCTCTGTCTCTCTCTCTCCCCCCCCCCCTTTCTTTTTTTTTTTTA|TTAACGCCTCTCACACTCTCACCCACGCCGGAGAGCGGTGGC
hr15:58207591-58207637 + E=7e-05
ACCATCCCACCGCACTGCC
```
